# Asymptomatic fetal bone retention detected 12 years after termination of pregnancy: case report

**DOI:** 10.11604/pamj.2023.46.100.39910

**Published:** 2023-12-12

**Authors:** İbrahim Kale, Cumhur Selçuk Topal

**Affiliations:** 1 Department of Obstetrics and Gynecology, Umraniye Training and Research Hospital, Istanbul, Turkey,; 2 Department of Pathology, Umraniye Training and Research Hospital, Istanbul, Turkey

**Keywords:** Abortion, curettage, intrauterine device, pregnancy termination, case report

## Abstract

Intrauterine retention of fetal bone fragments is a rare complication that can be seen after pregnancy termination, especially in advanced gestational weeks. Here, we present a case of intrauterine fetal bone retention detected during routine gynecological examination in an asymptomatic woman whose pregnancy was terminated 12 years ago. Under local anesthesia and ultrasound guidance, the fetal bone was removed with a grasper. This case report highlights the importance of post-curettage ultrasound examination to ensure that no fetal tissue is left behind after termination of pregnancy.

## Introduction

Intrauterine bone retention is a rare complication of pregnancy termination in the second or third trimester. Very rarely, the bone may be formed by a secondary metaplastic process associated with chronic inflammation in the endometrium [1,2]. The residual fetal bone may cause dysfunctional uterine bleeding, dysmenorrhea, vaginal discharge, pelvic inflammatory disease [3], chronic pelvic pain [4] or infertility [5,6]. Here, we present an asymptomatic patient with incidentally detected intrauterine fetal bone retention.

## Patient and observation

**Patient information:** a 47-year-old woman with gravida 7, parity 4, living 2, curettage 3 obstetric history was referred to our hospital for the removal of a part of the intrauterine device (IUD) remaining in the uterus. While she did not have any complaints, an echogenic glowing image in the uterus was observed in the transvaginal ultrasound (TVUSG) examination in the routine gynecological control at an external center, and it was thought that this may be a part of the IUD. According to the anamnesis given by the patient, the patient voluntarily terminated her 14-week pregnancy 12 years ago. One month after the termination of her pregnancy, she had an IUD application at the family medicine center and was protected with IUD for 10 years. After her IUD expired, she had her IUD removed at the family health center 2 years ago. The patient, who did not have any complaints within these 12 years, did not apply for a gynecological examination.

**Clinical findings:** the patient´s general body examination was normal and vital signs were within normal limits. In the sterile speculum examination performed as part of the gynecological examination, the vagina was normal, the cervix was multiparous, and there was no bleeding or discharge.

**Timeline of the episode:** January 2021: an echogenic glowing image in the uterus was observed in the ultrasound at an external center. February 2021: the intrauterine foreign body was removed with a grasper and the patient was discharged after the procedure. March 2021: the pathology report came in accordance with the fetal bone.

**Diagnostic assessment:** on TVUSG examination, the uterus antevert, myometrium heterogeneous, endometrium thickness was 5 mm and both ovaries were in normal appearance. A linear echogenic body approximately 15.5 mm long was observed in the endometrial cavity. It was thought that this foreign body was a part of the IUD that the patient had used before.

**Figure 1:**
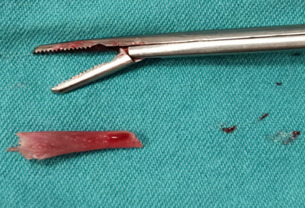
macroscopic view of fetal bone removed from the uterus

**Therapeutic intervention:** under local anesthesia, the foreign body was removed by entering the endometrial cavity with a grasper under suprapubic ultrasound guidance. A hard, 1.5 cm foreign body was sent for pathological examination (Figure 1).

**Figure 2:**
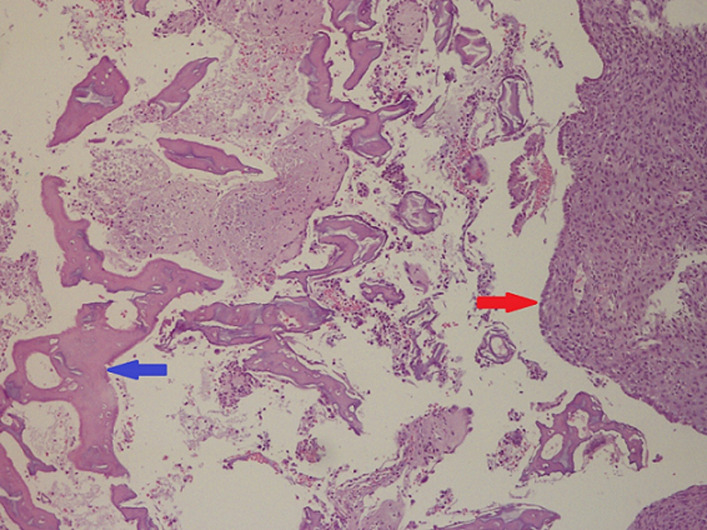
decidualized endometrium with fetal bone, the blue arrow indicates fetal bone and the red arrow indicates decidualized endometrium (100 x H&E)

**Follow-up and outcomes:** the patient tolerated the procedure easily and was discharged on the same day. The pathology report came in accordance with the fetal bone (Figure 2).

**Patient perspective:** it was quite surprising that I lived with a piece of fetal bone in my uterus for 12 years without any complaints. I am lucky that it was removed with a simple procedure in the outpatient department.

**Informed consent:** written informed consent was obtained from the patient for this case report.

## Discussion

In our country, IUDs are generally applied and removed in family health centers, and ultrasound examination is not performed before or after these procedures. The patient mentioned here also had an IUD application in the family health center one month after her pregnancy termination but did not have any gynecological ultrasound examination before or after this procedure. The patient, who did not have any complaints, lived with fetal bone in her uterus for 12 years. A systematic review published in 2016 on patients with endometrial bone or bone fragments found that infertility was the most common symptom at 52%, while only 5% of patients were asymptomatic. While 65.4% of these cases were diagnosed by ultrasound, 15.4% by dilatation and curettage, and 11% by hysterosalpingography, hysteroscopy was used for diagnosis in only 1.2% [2]. It has been shown that fetal bone can stimulate the synthesis of endometrial prostaglandins [7] which may explain the cause of dysmenorrhea, and chronic pelvic pain [4]. The retained bone fragments also may create an ideal environment for bacterial colonization and cause recurrent purulent vaginal discharge. In some cases, this causes severe attacks of pelvic inflammatory disease that require bilateral adnexectomy and total hysterectomy [3].

With the combination of foreign body effects and increased endometrial prostaglandins, the remaining fetal bone can act as an IUD and cause secondary infertility. Secondary infertile patients were reported to achieve pregnancy after the remaining fetal bone was removed [5,6]. Khan *et al*. stated that 82.2% of the secondary infertile patients with fetal bone remaining can conceive spontaneously after the removal of the intrauterine bone [2]. Unlike all the symptomatic cases mentioned above, the case discussed here lived asymptomatically with the fetal bone remaining for 12 years. Since she did not have fertility desire or any complaints, the patient hasn´t seen any specialist for her routine gynecologic examination.

## Conclusion

In conclusion, this case report highlights the importance of gynecological ultrasound examination after the pregnancy termination. Clinicians should not forget about fetal bone retention, which is a rare complication after the termination of pregnancy, especially in the advanced weeks.
